# PW03-018 – Efficacy of Anakinra in recurrent pericarditis

**DOI:** 10.1186/1546-0096-11-S1-A244

**Published:** 2013-11-08

**Authors:** M Finetti, A Insalaco, L Cantarini, M D'Alessandro, A Meini, L Breda, M Alessio, P Picco, A Martini, M Gattorno

**Affiliations:** 1 Pediatric Reumathology Second Division, IRCCS G. Gaslini, Genoa, Italy; 2 Ospedale Pediatrico Bambino Gesù, Rome, Italy; 3 Policlinico Le Scotte, Università di Siena, Siena, Italy; 4 Spedali Civili di Brescia, Brescia, Italy; 5 Ospedale Policlinico di Chieti, Chieti, Italy; 6 Università di Napoli Federico II, Napoli, Italy

## Introduction

Recurrent pericarditis represents an important complication of acute pericarditis. Therapeutic approach during recurrences consists of NSAID administration. However steroid is often necessary to control disease flares. IL-1 inhibitors efficacy has been anecdotally described as effective in the control of the disease in steroid-dependent and Colchicine-resistant patients.

## Objectives

To evaluate the long term response to treatment with Anakinra (IL-1 receptor antagonist) in multicenter cohort of patients affected by idiopathic recurrent pericarditis.

## Methods

Fifteen patients (12 pediatrics and 3 adults; M:F=11:4) affected by idiopathic recurrent pericarditis and followed by 6 different national referral centers were enrolled in the study. The mean age was 22 years (range 9-60 yrs); mean age at onset was 16 years (5-49 yrs), mean age at the beginning of treatment was 19 years (6-56 yrs). All patients received an initial dosage of 1-2 mg/Kg/die. All the patients presented steroid-dependence and 14 of them had received Colchicine during history disease. Outcomes evaluated in our study were i)response to Anakinra, defined as resolution of pericardial symptoms associated to normalization of laboratory-instrumental findings after first administration of the drug; ii)long term remission during IL-1 receptor antagonist regimen defined as absence of relapses during monotherapy; iii)resolution after Anakinra discontinuation.

## Results

All the patients that received Anakinra during active disease (13 pts) presented a dramatic therapeutic response featured by a very rapid disappearance of precordial pain, fever, rub and normalization of acute phase reactants within a few hours from drug administration. Continuous therapy allowed rapid tapering and then discontinuation of steroid, Colchicine and NSAID administration. During continuous daily treatment (mean FU=11 months, range 5-17 months), no patient presented a relapse of the disease; 14 patients started tapering and 8 of them experienced a relapse (mean time since tapering start to relapse=9 months, range 2-17 months). In all patients, disease flare was successfully and quickly controlled by daily full-dose administration of Anakinra, without the requirement of any steroid treatment. A total of 10 flares have been observed in these 8 patients. In 5 patients Anakinra was successfully discontinued after 24 months of treatment (range 17-32 months). The mean time of remission since the withdrawn of the drug is now 12 months (range 2-24 months). At the last follow-up all patients were in remission. Two patients are still receiving daily administration of Anakinra as monotherapy. In 8 patients Anakinra tapering is ongoing.

## Conclusion

The long term use of Anakinra in monotherapy is associated to a persistent control of clinical-laboratoristic-instrumental features of idiopathic recurrent pericarditis. In almost 50% of the patients reactivation of clinical manifestations during Anakinra tapering was observed.

## Disclosure of interest

None declared

